# The Incremental Yield of CMA Over Karyotype in Fetal Growth Restriction—A Systematic Review and Meta‐Analysis

**DOI:** 10.1002/pd.70022

**Published:** 2025-11-14

**Authors:** Ioakeim Sapantzoglou, Evangelia Kontogeorgi, Vasilios Pergialiotis, Konstantinos Tasias, Angeliki Rouvali, Afroditi Pegkou, Marianna Theodora, Georgios Daskalakis, Panagiotis Antsaklis

**Affiliations:** ^1^ 1st Department of Obstetrics and Gynecology Alexandra Hospital National and Kapodistrian University of Athens Athens Greece

## Abstract

The main objective of our study was to conduct a systematic literature review and a meta‐analysis to evaluate the incremental yield of chromosomal microarray analysis compared with karyotyping in cases of fetal growth restriction. Our review was designed according to the PRISMA guidelines. It included all observational studies that reported the results of CMA testing in fetuses diagnosed with growth restriction without additional findings (isolated FGR), with structural abnormalities (malformed FGR) and with the presence of additional findings that would not qualify as structural abnormalities (nonmalformed FGR). The study included 22 studies with a total of 2275 cases of affected fetuses that met the inclusion criteria for analysis. Combined data from these studies revealed an overall 3% incremental yield of CMA over karyotyping (95% CI 2%–5%) in isolated cases, an overall 4% incremental yield of CMA over karyotyping (95% CI 3%–5%) in nonmalformed FGR cases and an overall 10% incremental yield (95% CI –13%) in malformed FGR cases. Our findings may be useful in clinical practice to guide management options and the counseling of the couples to individualize patient care and facilitate clinicians when they come across such a common clinical entity.

## Summary

- What is already known about this topic?
  ◦ Underlying chromosomal abnormalities represent one of the most common causes of FGR and while karyotyping has demonstrated efficacy in identifying significant structural mutations, its inability to detect copy number variants restricts the potential identification of pathological submicroscopic DNA gains or losses.
- What does this review add?
  ◦ CMA demonstrated an overall 3% incremental yield over karyotyping in FGR cases without any additional findings, an overall 4% incremental yield in nonmalformed FGR cases and an overall 10% incremental yield in malformed FGR cases.

## 1 Introduction

Fetal growth restriction (FGR) is well known to be associated with an increased likelihood of subsequent adverse perinatal outcomes as well as a high risk of perinatal morbidity and mortality [1, 2]. It is well established that uteroplacental insufficiency is one of the most prevalent etiologies of abnormal growth, but several additional maternal, placental and fetal factors have been identified as potential causes [3, 4]. Underlying chromosomal aberrations represent one of the most common fetal causes of FGR and may constitute up to 19% of affecting fetuses with their incidence varying and being dependent upon the gestational age at diagnosis and the coexistence of fetal structural abnormalities [5, 6, 7]. In this view, while karyotyping has demonstrated efficacy in identifying significant structural mutations, polyploidies and aneuploidies, it confers limitations such as the inability to detect copy number variants (CNVs), restricting as such the potential identification of pathological submicroscopic gains or losses of DNA. Over the recent years, chromosomal microarray analysis (CMA), which is a molecular approach that identifies copy number variants with a resolution of 10 Kb or more, has emerged and introduced as a necessary and extended tool of the prenatal assessment [8].

A systematic review and meta‐analysis by Borell et al. [9] demonstrated an incremental yield of 10% and 4% of CMA over standard karyotyping in cases of FGR fetuses with and without additional structural abnormalities, respectively. However, the nonmalformed FGR group included cases with additional ultrasound findings such as soft markers of aneuploidy and amniotic fluid abnormalities, creating such a potentially heterogeneous subgroup. Given the fact that soft markers continue to be utilized in the screening for trisomy 21 and that fetal evaluation utilizing soft markers seems to be effectively aiding the detection of additional genetic abnormalities, including CNVs [10], the true incidence of CNVs in truly isolated FGR cases remains under question.

As such, the aim of our study is,first, to update the results of the previous meta‐analysis regarding FGR fetuses with additional structural abnormalities (malformed FGR) and second, to investigate the incremental yield of CMA over standard karyotyping in detecting underlying genetic aberrations in truly isolated FGR (isolated FGR) and in cases with coexistent soft signs or amniotic fluid volume abnormalities (nonmalformed FGR).

## 2 Methods

This systematic review and meta‐analysis was designed according to the Preferred Reporting Items for Systematic Reviews and Meta‐Analyses (PRISMA) guidelines as well as the MOOSE Guidelines for Meta‐Analyses and Systematic Reviews of Observational Studies. This review was registered in the PROSPERO international database for systematic reviews (reference: CRD420251082633).

### 2.1 Eligibility Criteria

The present systematic review included all observational studies (prospective/retrospective cohort, case–control, nested case–control, and cross‐sectional) that reported the results of CMA testing in fetuses diagnosed with growth restriction without additional findings (isolated FGR), with structural abnormalities (malformed FGR) and with the presence of additional findings that would not qualify as structural abnormalities (soft signs, Doppler or amniotic fluid volume abnormalities—nonmalformed FGR). Case reports, small case series, letters to the editor, animal studies, and review articles were not included. Conference proceedings and abstracts were also planned to be excluded, as they lack important information that is necessary for the assessment of study limitations and quality of evidence.

### 2.2 Information Sources and Search Strategy

**FIGURE 1:**
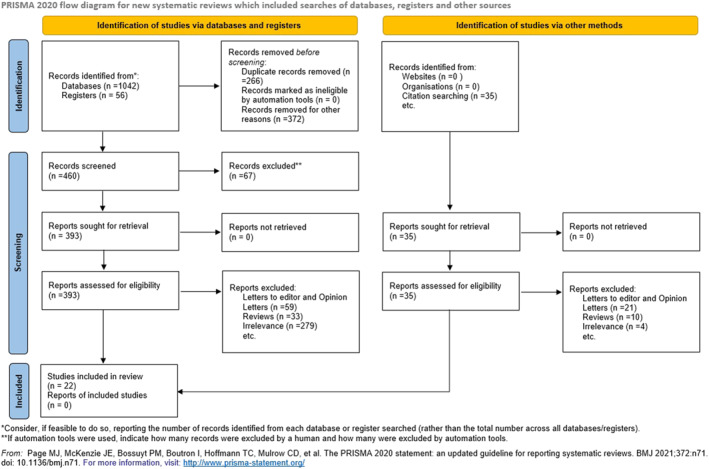
Search strategy.

The Medline (1966–2025), Scopus (2004–2025), Clinicaltrials.gov (2008–2025), EMBASE (1980–2025), Cochrane Central Register of Controlled Trials CENTRAL (1999–2025), and Google Scholar (2004–2025) databases were used in our primary search, along with the reference lists of electronically retrieved full‐text papers. The date of our last search was set at 31 March 2025. Our search strategy included the text words “fetal growth restriction” or “prenatal growth restriction” or “intrauterine growth restriction” or “ultrasound anomaly” and “array comparative genomic hybridization” or “copy number variation” and is briefly presented in Figure 1. The main search algorithm was as follows: (“fetal growth restriction” OR “prenatal growth restriction”OR “intrauterine growth restriction” OR “ultrasound anomaly”) AND (“array comparative genomic hybridization” OR “aCGH” OR “copy number variation”). The search identified 1133 potentially relevant studies, but 1111 were excluded because they were non‐relevant articles, reviews, opinion letters, or letters to the editor, or we were unable to retrieve the available data after contacting the authors. Thus, in total, only 22 peer‐reviewed papers were considered for inclusion in our systematic review and in the current meta‐analysis.

### 2.3 Study Selection

The study selection process involved three consecutive stages. First, the titles and abstracts of all electronic papers were screened to assess their potential eligibility. Subsequently, all articles that met or were presumed to meet the eligibility criteria were retrieved as full texts. Finally, all observational (both prospective and retrospective) studies that reported the results of CMA testing in fetuses diagnosed with isolated FGR, nonmalformed FGR and malformed FGR were deemed eligible. Study selection was performed by two authors independently, while any potential discrepancies were resolved through their consensus (Figure 1).

### 2.4 Data Collection

The following data were extracted from each included study: name of first author, year of publication, study design, recruitment period, inclusion and exclusion criteria, FGR definition, malformed FGR definition (where applicable), nonmalformed FGR definition (where applicable), the inclusion criteria for the performance of CMA and the type of CMA utilized. The soft signs mentioned and included in the studies encompassed the following.

- aberrant right subclavian artery (ARSA)
- persistent left superior vena cava (PSVC)
- single umbilical artery (SUA)
- echogenic bowel
- urinary pelvis dilatation
- echogenic kidneys
- mild pericardial effusion
- increased nuchal fold
- cardiomegaly/right‐sided cardiac dominance (without evidence of cardiac structural anomalies)
- persistent right umbilical vein (PRUV)
- short long bones (without evidence of skeletal dysplasia)
- echogenic cardiac focus
- choroid plexus cysts
- absent nasal bone
- talipes
- sandal gap
- fifth finger clinodactyly
- ambiguous genital
- absent nasal bone
- retrognathia.

When important data were missing, attempts were made to contact the corresponding authors. Data extraction was performed by three authors, and any possible disagreements were resolved through consensus or by discussion with all authors. The methodological characteristics of the included studies are depicted in Table 1.

**TABLE 1:** Methodological characteristics of the included studies.

Author, year	Type of study	Recruitment period	Inclusion criteria	Exclusion criteria	FGR definition	Malformed FGR definition	Nonmalformed FGR definition	Inclusion criteria for CMA	CMA type
Chen X., 2024 [11]	Retrospective observational study	October 2022– December 2023	Ultrasound anomalies with invasive prenatal diagnostic indications Detailed genetic counseling and informed consent No contraindications for invasive prenatal diagnosis	Contraindications for invasive testing	EFW < 10th centile	NA	Increased NT Amniotic fluid volume abnormalities	All included fetuses	Affymetrix standard procedures (affymetrix cytoscan 750K array chip)
Nguyen P.T., 2025 [12]	Cross‐sectional descriptive‐ retrospective observational study	January 2021– December 2023	FGR with additional anomalies	NR	EFW < 10th centile	Cardiac anomalies Urogenital anomalies Gastrointestinal anomalies Skeletal anomalies CNS anomalies	NA	All included fetuses	Affymetrix GeneChip 3000 dx v2 system: 50 and 200 kB
Elron E., 2024 [13]	Retrospective case series	January 2017–February 2023	Fetuses with additional major or minor fetal anomalies, FGR and polyhydramnios	Major fetal malformation detected prior to the third trimester Presence of soft signs Possible maternal contamination or technical failures Amniocentesis performed because of a suspected CMV infection	EFW < 10th centile or AC < 5th centile	NA	NA	All included fetuses	CytoScan 750K array
Peng C., 2024 [14]	Cohort study	October 2014–2021	Fetuses with FGR with or without additional ultrasound abnormalities	NR	EFW < 10th centile or AC < 10th centile	Cardiac anomalies CNS anomalies Facial anomalies Urogenital anomalies Abdominal wall anomalies Gastrointestinal anomalies Skeletal anomalies	ARSA PLSVC Single umbilical artery Echogenic bowel Urinary pelvis dilatation Echogenic cardiac focus Amniotic fluid volume abnormalities	All included fetuses	The affymetrix CytoScan 750 K array (Thermo, USA)
Dap M., 2022 [15]	Retrospective observational study	January 2013– December 2020	Fetuses diagnosed with FGR without structural anomalies before 24 weeks of gestational age	Multiple pregnancies Presence of structural fetal malformation Imprecise pregnancy dating Suspected congenital infections	EFW < 3^rd^ centile	NA	NA	All included fetuses	Human genome CGH micro array 180 k (Agilent), algorithm ADM2
Zhou H., 2022 [16]	Retrospective observational study	January 2016– December 2020	Fetuses diagnosed with FGR Reliable pregnancy dating	Multiple pregnancies Presence of structural malformation Suspicion of TORCH infection	EFW < 3^rd^ centile	NA	ARSA PLSVC Single umbilical artery Echogenic bowel Echogenic kidneys Mild pericardial effusion	All included fetuses	Affymetrix CytoScan HD/750K array
Monier I., 2021 [17]	Retrospective observational study	1^st^ January 2016–31 December 2016	Singleton fetuses diagnosed with isolated FGR	Multiple pregnancy Imprecise pregnancy dating < 18 years old, Presence of malformation	EFW < 10th centile	NA	NA	All included fetuses	Pre cytoNEM, (resolution: 105 K genome‐wide randomized probes with specific enrichment) SurePrint G3 Hu man (resolution: 60e180 K genome‐wide randomized probes; agilent) OmniExpress 24 design (resolution: 130e400 millions of genome‐wide randomized clusters)
Hui A.S., 2020 [18]	Retrospective cohort study	July 2007–aug 2017	Fetuses with abnormal ultrasound findings restricted to a single anatomic system or presence of nonspecific findings and normal karyotype results	Pregnancies with soft markers only	NR	NA	NA	All included fetuses	Customized fetal DNA chip version 1.2 (44K, agilent Technologies inc., Santa clara, CA, USA) or Fetal DNA chip version 2.0 (60K)
An G., 2018 [19]	Retrospective cohort study	July 2015– February 2018	FGR singleton fetuses without additional ultrasound findings	Presence of additional structural findings Abnormal NIPT result Multiple pregnancies Chronic renal disease Preeclampsia, Antiphospholipid syndrome TORCH infection	EFW < 10th centile	NA	NA	All included fetuses	CytoScan 750 K array
Peng R., 2017 [20]	Retrospective cohort study	2013–2017	Singleton or dichorionic twins FGR diagnosis with or without amniotic fluid abnormalities	Monochorionic twins Presence of additional structural abnormalities	EFW < 10th centile	NA	NA	All included fetuses	Affymetrix CytoScan HD array
Brun S., 2018 [21]	Retrospective cohort study	January 2012– December 2017	FGR fetuses with or without additional structural findings	NR	EFW < 3rd centile	Cardiac anomalies CNS anomalies Facial anomalies Urogenital anomalies Skeletal anomalies		Fetuses with a normal FISH result	G2505 C scanner
Xia M., 2020 [22]	Retrospective observational study	January 2016– December 2019	Fetuses with ultrasound abnormalities Presence of soft signs Presence of non‐structural abnormalities such as FGR, polyhydramnios/oligohydramnios Abnormal NIPT result History of adverse pregnancy outcomes	NR	NR	NA	NA	All included fetuses	CytoScan Reagent Kit Affymetrix CytoScan 750 K array
Borell A., 2016 [23]	Prospective observational study	January 2009–July 2015	• FGR diagnosis < 32 weeks of gestational age	Fetuses with known aneuploidies	EFW < 3rd centile	Cardiac anomalies CNS anomalies Facial anomalies Urogenital anomalies Ascites	Amniotic fluid volume abnormalities Prominence of cerebral sulci Increased NT Retrognathia Cardiomegaly/Right cardiac dominance ARSA Muscular VSD Echogenic bowel Bowel dilatation Pericardial effusion Ambiguous genitalia Talipes	Fetuses with a normal QF‐PCR	CytoChip focus constitutional (BlueGenome, illumina) BAC microarray: 100 kb
Srebniak, 2016 [24]	Prospective observational study	2009–2013	EFW < 10^th^ with	Fetuses with known aneuploidies or triploidies	EFW < 10th centile	Cardiac anomalies CNS anomalies Facial anomalies Urogenital anomalies Ascites/pleural effusion/cystic hygroma/hydrops Gastrointestinal anomalies Skeletal anomalies Increased NT > 3.5 mm Presence of second trimester soft signs Multiple anomalies Intrauterine fetal death	NA	Fetuses with a normal karyotype	HumanCytoSNP‐12 array (illumina): 300k SNP array platform
De wit M.C., 2016 [25]	Retrospective cohort study	September 2011–May 2015	All singleton pregnancies with an AC ≤ 5th centile between 18 and 24 weeks of gestational age	Cases with structural fetal malformations	AC ≤ 5th centile	NA	Single umbilical artery Echogenic bowel Urinary pelvis dilatation Shortened long bones Echogenic cardiac focus Choroid plexus cysts Amniotic fluid volume abnormalities	Fetuses with a normal QF‐PCR	HumanCytoSNP‐12 array (illumina): 300k SNP array platform
Lovrecic L., 2016 [7]	Retrospective observational study	July 2012– October 2015	Abnormal chromosomal rearrangement in a family member or prior pregnancy Ultrasound anomalies Known abnormal fetal karyotype	NR	NR	Urogenital anomalies Cystic hygroma	NR	Fetuses with a normal QF‐PCR	Agilent SurePrint G3 Unrestricted array‐CGH ISCA v2 8x 60k: 100 kb
Zhu H., 2016 [26]	Retrospective cohort study	Mar 2013–October 2015	FGR with ultrasound abnormalities FGR with a positive first or second trimester screening test FGR with advanced maternal age FGR with suspected congenital infection Isolated FGR excluding maternal causes	NR	EFW < 10th centile	Cardiac anomalies CNS anomalies Skeletal anomalies Urogenital anomalies Gastrointestinal anomalies	Amniotic fluid volume abnormalities Doppler abnormalities	All fetuses	CytoScan HD array (affymetrix): 100 kb
Oneda B., 2014 [27]	Retrospective observational study	August 2010–April 2013	Advanced maternal age Anomalies detected on ultrasound Increased NT Prior history of genetic disorder Abnormal maternal serum screening Parental concern	NR	NR	NA	NR	Fetuses with a normal karyotype	Whole Genome2.7 M array/Cytoscan HD array (genome wide resolution of 20–100kb)
Shaffer L.G., 2012 [28]	Retrospective observational study	July 2004– December 2011	Fetuses with abnormal ultrasound findings, including soft markers.	Fetuses with known abnormal karyotype Family history of a chromosome rearrangement in a parent Fetal demise	NR	Cardiac anomalies CNS anomalies Skeletal anomalies Urogenital anomalies Gastrointestinal anomalies Facial anomalies Respiratory anomalies Cystic hygroma/Hydrops/Increased NT Multiple anomalies	Amniotic fluid volume abnormalities Choroid plexus cysts Echogenic foci in the heart Isolated short long bones Absent nasal bone Single umbilical artery Persistent right umbilical vein Sandal gap Fifth finger clinodactyly	All fetuses	‘SignatureChip whole Genome (coppinger)
Gruchy N., 2011 [29]	Retrospective observational study	September 2009– December 2010	Fetuses with presence of isolated FGR FGR associated with ultrasound malformation(s) At least two ultrasound malformations in two different organs	Aneuploidies such as trisomies 13, 18, 21, 45XO, or deletion 22q11.2 syndrome	NR	NR	NA	Fetuses with a normal karyotype and normal FISH for 22q11.2 deletion	CytoChip focus slides (BlueGnome):100kb
Kleeman L., 2009 [30]	Retrospective observational study	April 2007–November 2008	Fetuses with significant structural malformations and/or FGR	Fetuses with only “soft markers” for aneuploidy	• EFW < 10^th^ centile	Cardiac anomalies CNS anomalies vSkeletal anomalies Urogenital anomalies Cystic hygroma/Hydrops Multiple anomalies	NA	Fetuses with a normal karyotype	Signature chip 4.0 or Signature WG chip
Van den Veyver I.B., 2009 [31]	Retrospective observational study	September 2005–fer 2008	Advanced maternal age Anomalies detected on ultrasound Prior history of genetic disorder Abnormal maternal serum screening Parental concern	NR	NR	Polydactyly Hypoplastic left heart Micrognathia Micropenis Cystic hygroma	NR	Fetuses with a normal karyotype	Baylor college of medicine BAC chromosomal microarray V5 or 6 or V6 of the BCM oligonucleotide chromosomal microarray

Abbreviations: AC: abdomen circumference, ARSA: Aberrant right subclavian artery, CGH: comparative genomic hybridization, CMA: chromosomal microarray analysis, CMV: cytomegalovirus, CNV: Copy Number Variation, EFW: estimated fetal weight, FGR: Fetal growth restriction, NA: Not applicable, NIPT: non‐invasive prenatal testing, NR: Not reported, NT: nuchal translucency, PLSVC: persistent left superior vena cava, QF‐PCR: quantitative fluorescence polymerase chain reaction, SNP: single‐nucleotide polymorphism, WG: Whole Genome.

### 2.5 Quality Assessment

The methodological qualities of the included studies were assessed by two independent reviewers using the QUADAS‐2 (Quality Assessment of Diagnostic Accuracy Studies‐2) tool that evaluates the technique of patient selection, the indexed test, the reference standard to that test and the flow and timing of the test/study. When the two authors disagreed, a final consensus was given by a third reviewer (Table 2). The overall risk was decided based on the suggestions made by the QUADAS‐2 group.

**TABLE 2:** Quality assessment summary of the included studies using the Quality Assessment tool for Diagnostic Accuracy Studies (QUADAS‐2) checklist.

	Patient selection					Index TEST(S)				Reference standard				Flow and timing				
Author, year	Was a consecutive or random sample of patients enrolled?	Was a case‐control design avoided?	Did the study avoid inappropriate exclusions?	Risk of bias	Concerns regarding applicability	Were the index test results interpreted without knowledge of the results of the reference standard?	If a threshold was used, was it pre‐specified?	Risk of bias	Concerns regarding applicability	Is the reference standard likely to correctly classify the target condition?	Were the reference standard results interpreted without knowledge of the results of the index test?	Risk of bias	Concerns regarding applicability	Was there an appropriate interval between index test(s) and reference standard?	Did all patients receive a reference standard?	Did patients receive the same reference standard?	Were all patients included in the analysis?	Risk of bias
Chen et al., 2024 [11]	No	Yes	Yes	No	Low	No	Yes	Low	Low	Yes	No	Low	Low	No	Yes	Yes	Yes	Low
Nguyen et al., 2025 [12]	Unclear	Yes	Yes	Unclear	Low	No	Yes	Low	Low	Yes	No	Low	Low	No	Yes	Yes	Yes	Low
Elron et al., 2024 [13]	Unclear	Yes	Yes	Unclear	Low	No	Yes	Low	Low	Yes	No	Low	Low	No	Yes	Yes	Yes	Low
Peng et al., 2024 [14]	Unclear	Yes	Yes	Unclear	Low	No	Yes	Low	Low	Yes	No	Low	Low	No	Yes	Yes	Yes	Low
Dap et al., 2022 [15]	No	Yes	Yes	No	Low	No	Yes	Low	Low	Yes	No	Low	Low	No	Yes	Yes	Yes	Low
Zhou et al., 2022 [16]	Unclear	Yes	Yes	Unclear	Low	No	Yes	Low	Low	Yes	No	Low	Low	No	Yes	Yes	Yes	Low
Monier et al., 2021 [17]	No	Yes	Yes	No	Low	No	Yes	Low	Low	Yes	No	Low	Low	No	Yes	No	Yes	Low
Hui et al., 2020 [18]	No	Yes	Yes	No	Low	No	Yes	Low	Low	Yes	No	Low	Low	No	Yes	No	Yes	Low
An et al., 2018 [19]	No	Yes	Yes	Unclear	Low	No	Yes	Low	Low	Yes	No	Low	Low	No	Yes	Yes	Yes	Low
Peng et al., 2017 [20]	No	Yes	Yes	Unclear	Low	No	Yes	Low	Low	Yes	No	Low	Low	No	Yes	Yes	Yes	Low
Brun et al., 2018 [21]	No	Yes	Yes	Unclear	Low	No	Yes	Low	Low	Yes	No	Low	Low	No	Yes	Yes	Yes	
Xia et al., 2020 [22]	No	Yes	Yes	No	Low	No	Yes	Low	Low	Yes	No	Low	Low	No	Yes	Yes	Yes	Low
Borell et al., 2016 [23]	Unclear	Yes	Yes	Unclear	Low	No	Yes	Low	Low	Yes	No	Low	Low	No	Yes	Yes	Yes	Low
Srebniak et al., 2016 [24]	No	Yes	Yes	No	Low	No	Yes	Low	Low	Yes	No	Low	Low	No	Yes	Yes	Yes	Low
De wit et al., 2016 [25]	Unclear	Yes	Yes	Unclear	Low	No	Yes	Low	Low	Yes	No	Low	Low	No	Yes	No	No	Low
Lovrecic et al., 2016 [7]	No	Yes	Yes	No	Low	No	Yes	Low	Low	Yes	No	Low	Low	No	Yes	No	Yes	Low
Zhu et al., 2016 [26]	Unclear	Yes	Yes	Unclear	Low	No	Yes	Low	Low	Yes	No	Low	Low	No	Yes	Yes	Yes	Low
Oneda et al., 2014 [27]	Unclear	Yes	Yes	Unclear	Low	Yes	Yes	Low	Low	Yes	Yes	Low	Low	No	Yes	No	Yes	Low
Shaffer et al., 2012 [28]	Unclear	Yes	Yes	Unclear	Low	No	Yes	Low	Low	Yes	No	Low	Low	No	Yes	Yes	Yes	Low
Gruchy et al., 2011 [29]	Yes	Yes	Yes	Unclear	Low	No	Yes	Low	Low	Yes	No	Low	Low	No	Yes	Yes	Yes	Low
Kleeman et al., 2009 [30]	Unclear	Yes	Yes	Unclear	Low	No	Yes	Low	Low	Yes	No	Low	Low	No	Yes	No	Yes	Low
Van den Veyver et al., 2009 [31]	Unclear	Yes	No	Unclear	Low	No	Yes	Low	Low	Yes	No	Low	Low	No	Yes	No	Yes	Low

### 2.6 Data Synthesis

The incremental yield (risk difference) of CMA was defined as the yield beyond karyotyping for each prenatal series. The incremental yield was calculated as the ratio of undetected aberrant results by karyotyping (CNV < 10 Mb at microarray analysis) to the total number of cases with a normal karyotype. Variants of uncertain significance were excluded from this study.

Risk differences were consolidated using inverse variance weighting to estimate the overall and stratified CMA incremental yield through RStudio (RStudio Inc., Boston, MA). Subsequently, corresponding forest plot graphs were generated. Confidence intervals (CIs) were computed. The Higgins I^2^ test was employed to evaluate statistical heterogeneity. Due to the limited statistical power of heterogeneity tests, we established statistically significant heterogeneity as a Cochran Q test with *p* < 0.1 or I^2^ > 30%. A random‐effects model was utilized due to substantial heterogeneity.

## 3 Results

Our search identified 1133 potentially relevant studies, but 1111 were excluded after reviewing the titles and the abstracts and after the exclusion of non‐relevant articles, case reports, opinion letters, reviews and letters to the editor. Overall, 22 studies were included in the present systematic review (19 retrospective, 2 prospective cohort studies and 1 retrospective case series) that enrolled a total of 2275 cases of patients [7, 11, 12, 13, 14, 15, 16, 17, 18, 19, 20, 21, 22, 23, 24, 25, 26, 27, 28, 29, 30, 31]. The search strategy is briefly presented in Figure 1.

The methodological characteristics of the included studies are presented in Table 1 and include the inclusion and exclusion criteria, the FGR definition, the associated structural anomalies that define FGR cases as malformed, the associated findings that define FGR as nonmalformed, the criteria for the performance of CMA and the type of microarray used in each study. FGR definition was provided in 15 studies while the remaining 7 studies did not provide any specific criterion for FGR definition [7, 18, 22, 27, 28, 29, 31]. The cutoffs used were the 10th percentile for the estimated fetal weight (EFW) in 8 studies [11, 12, 17, 19, 20, 24, 26, 30], the third percentile for the estimated fetal weight in 4 studies [15, 16, 21, 23], the fifth percentile for the abdominal circumference (AC) in 1 study [25], either the 10th percentile for EFW or the fifth percentile for AC in 1 study [13] and either the 10th percentile for EFW or the 10th percentile for AC in 1 study [14]. Twenty studies included CMA data from isolated cases of FGR [7, 11, 13, 14, 15, 16, 17, 18, 19, 20, 21, 22, 23, 25, 26, 27, 28, 29, 30, 31], 2 studies included data only from malformed cases of FGR [12, 24], 8 studies included data from both isolated and malformed cases [7, 20, 21, 23, 26, 27, 28, 29], 3 studies included data from both isolated and nonmalformed cases [11, 16, 25] and 3 studies included data from isolated, malformed and nonmalformed cases [14, 20, 23].

CMA was performed after a normal result at karyotyping in 5 studies [24, 27, 29, 30, 31], after normal quantitative fluorescence polymerase chain reaction (QF‐PCR) results in 3 studies [7, 23, 25], after normal Fluorescent in Situ Hybridization (FISH) in 1 study [21] and simultaneously to karyotyping in 13 studies [11, 12, 13, 14, 15, 16, 17, 18, 19, 20, 22, 26, 28].

**FIGURE 2:**
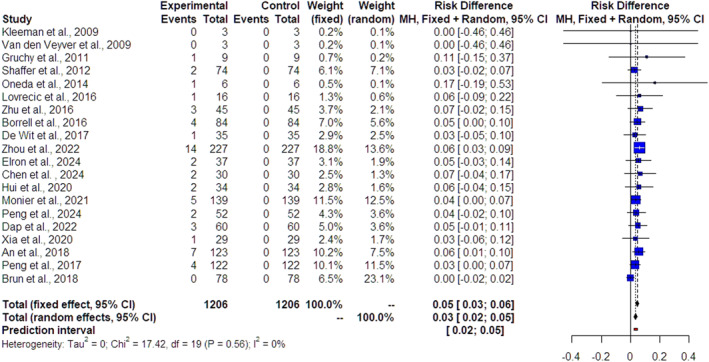
Forest plot of incremental yield by chromosomal microarray analysis (CMA) over karyotyping in isolated growth‐restricted fetuses with 95% confidence intervals (CIs) and weighted pooled summary statistics using a bivariate random‐effect model. Forest plot analysis: Vertical line = “no difference” point between the two groups. Red squares = Incremental yield of CMA over karyotyping of individual studies; Diamond = pooled incremental yield of CMA over karyotyping and 95% CI for all studies; Horizontal black lines = 95% CI; Horizontal red line = prediction intervals.

**FIGURE 3:**
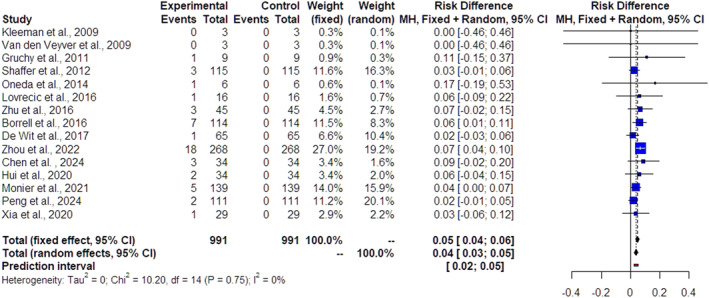
Forest plot of incremental yield by chromosomal microarray analysis (CMA) over karyotyping in nonmalformed growth‐restricted fetuses with 95% confidence intervals (CIs) and weighted pooled summary statistics using a bivariate random‐effect model. Forest plot analysis: Vertical line = “no difference” point between the two groups. Red squares = Incremental yield of CMA over karyotyping of individual studies; Diamond = pooled incremental yield of CMA over karyotyping and 95% CI for all studies; Horizontal black lines = 95% CI; Horizontal red line = prediction intervals.

**FIGURE 4:**
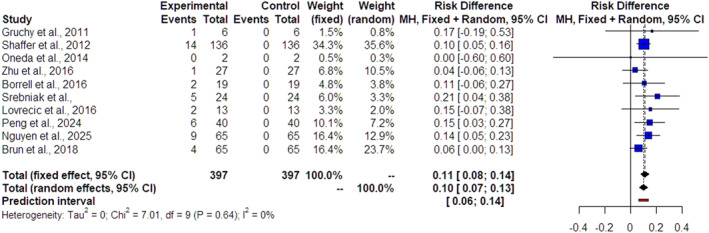
Forest plot of incremental yield by chromosomal microarray analysis (CMA) over karyotyping in malformed growth‐restricted fetuses with 95% confidence intervals (CIs) and weighted pooled summary statistics using a bivariate random‐effect model. Forest plot analysis: Vertical line = “no difference” point between the two groups. Red squares = Incremental yield of CMA over karyotyping of individual studies; Diamond = pooled incremental yield of CMA over karyotyping and 95% CI for all studies; Horizontal black lines = 95% CI; Horizontal red line = prediction intervals.

The forest plots of the 22 included studies and the pooled results from the meta‐analysis in cases of isolated FGR, nonmalformed FGR and malformed FGR are depicted in Figures 2, 3 and 4, respectively. The pooled data from the reviewed studies show an overall 3% incremental yield of CMA over karyotyping (95% CI 2%–5%, I^2^ = 0%) in isolated cases (Figure 2), an overall 4% incremental yield of CMA over karyotyping (95% CI 3%–5%, I^2^ = 0%) in nonmalformed FGR cases (Figure 3) and an overall 10% incremental yield (95% CI –13%, I^2^ = 0%) in malformed FGR cases (Figure 4). The observed incremental yield for each single study ranged from 0% to 17% in isolated cases, from 0% to 17% in nonmalformed FGR cases and from 0% to 21% in malformed FGR cases.

The clinically significant submicroscopic CNVs detected in nonmalformed FGR cases along with the additional findings of each case are presented in Table 3. The clinically significant submicroscopic CNVs detected in malformed FGR cases along with the structural anomalies identified in each case are presented in Table 4.

**TABLE 3:** Pathogenic and likely pathogenic CNVs and soft signs or additional findings identified by CMA analysis in fetuses with non‐malformed FGR and normal karyotype.

Author, year	Ultrasound findings	Pathogenic CNVs
Shaffer et al., 2012 [28]	NR	NR
Borrell et al., 2017 [23]	Oligohydramnios	Arr 3q29(195, 196, 032x2, 195,989, 448–196, 801, 209x1, 197, 225, 550x2)
	Polyhydramnios	Arr 22q11.22q11.23(22,691,548x2, 23,146,740–24,319,952x3, 25,254,126x2)
	Polyhydramnios	Arr 7q11.23(72,583,171x2, 72,699,013–73,955,362x1, 74,227,093x2)
Zhou et al., 2022 [16]	ARSA + Oligohydramnios	Arr [hg19] 18p11.32p11.21 (136226_15157836)x3
	ARSA, PLSVC	Arr [hg19] 4p16.3p16.1 (68345_8731855)x1
	Echogenic kidneys	Arr [hg19] 4p16.3p15.2 (68346_22565466)x1
	Mild pericardial effusion + Oligohydramnios	Arr [hg19] 13q33.3q34 (109549536_115107733)x1
	Hyperechogenic bowel	Arr [hg19] 21q11.2q22.3 (15041209_48097372)x3
Chen et al., 2024 [11]	NT thickening	NR

Abbreviations: ARSA: aberrant right subclavian artery, NT: nuchal translucency, NR: not reported, PLSVC: persistent left superior vena cava.

**TABLE 4:** Pathogenic CNVs and fetal anomalies identified by CMA analysis in fetuses with malformed FGR and normal karyotype.

Author, year	Ultrasound findings	Pathogenic CNVs
Srebniak et al., 2016 [24]	Cleft lip	arr[hg18] 4p16.3p16.1(38,283x2,38,283–8,321,040x1,8,321,040)x2dn
	Complex CHD	arr[hg18] 5q35.3(179,278,602x2,179286,688–180,638,145x1,180,857, 866x2)mat,16q24.2q24.3(86,358,459x2,86,364, 482–88,685,456x3,88,827,254)x2mat
	CCA, CHD, aberrant position lower leg, SUA	arr[hg18] 6q27(167,704,044x2,167,716,064–170,740,474x1,170,899, 992)x2mat,16p13.3p13.13(1x2,45,320–10,739,945x3,10,753,492)x2mat
	Thumb malformation, suspected cardiomyopathy	arr[hg18] 12p13.33(1x2,61,880–2,975,268x1,2,980,134)x2 x2dn,19p13.3p13.2(1x2,218,039–7,499,589x3,7,499,589x2)dn
	Microcephaly, CPC, increased NF, cardiac echogenic focus, club foot	arr[hg18] 22q11.21(19,3354,880x2,19,380,522–19,792,353x1,20,125, 656x2)mat
Lovrecic et al., 2016 [7]	Cystic hygroma	Arr 15q13.2q13.3(30,653,577–32,861,626)x3
	Multicystic kidney	Arr 16p12.2(21,837,492–22,407,931)x1
Peng et al., 2024 [14]	RAA with ALSA, VSD	arr[hg19] 2q24.1q31.1 (155,056,293–171,246,014) x1
	Abnormal facial morphology, ARSA, suspected hypospadias, and small renal measurements	arr[hg19] 4p16.3p15.31 (68,345–19,944,479)x1
	Atypical facial features	arr[hg19]4p16.3p15.33 (68,345–12,959,346)x1
	Small kindeys, SUA	arr[hg19]4p16.3p16.2 (68,345–5,612,162)x1
	Renal pelvis dilatation, the left heart was slightly smaller, the inner diameter of the suspected aortic arch was slightly narrower, the inner diameter of the coronary sinus was slightly wider, PLSVC	arr[hg19]Xq28 (153560563_153868487)x2
Nguyen et al., 2025 [12]	Ventriculomegaly, Cardiac echogenic focus	arr[GRCh38] 18p11.32(136227_1829674) x1
	Ureteral dilatation	arr[GRCh38] 4q34.1q35.2(175205219_189274455)x1
	ACC, absent CSP	arr[GRCh38] 16p13.3q24.3(35881_90088654)x3
	CPC, Hyperechogenic bowel	arr[GRCh38] 2q32.2q33.2(191099419_203580897)x1
	Ventriculomegaly	arr[GRCh38] 18q12.1q12.3(30592390_41322085)x1
	Pulmonary valve stenosis	arr[GRCh38] 22q11.21(18929330_21110475)x1.
	Complex CHD	arr[GRCh38] 4p16.3p15.32(68454_15939113)x1.
	Complex CHD	arr[GRCh38] 22q11.21(18147152_21110475)x1
	VSD	arr[GRCh38] 4p16.3p16.1(68454_8719854)x1
Brun et al., 2018 [21]	Lingual interposition	arr[hg19] 7q11.23(74139390_72726578)x1
	Microcephaly + Retrognathia	arr[hg19] 4p16.1p16.3(71552_7513828) x1,4p15.1p16.1(7582506_27840682)x3
	Small hyperechogenic kidneys, Retrognathia	Arr [hg19] 17q12 (34817422_36473234)x1 dn
	Facial dysmorphy, Complex CHD, echogenic bowel, Placentomegaly	arr[hg19] 7q33q36.3(137495879_159088636)x1dn, 10q25.1q26.3(109454712_135404523)x3 dn
	AVSD, large CSP, small CM	Arr [Hg19] 3q23q24(142467261_148447270)x1 mat
Gruchy et al., 2011 [29]	Exomphalos	Arr 7q11.23(72171274–74159511)x1
Zhu et al., 2016 [26]	Hypospadias, short nasal bone	Arr 2p25.3q37.3(15,702–242,755,901)x2
Shaffer et al., 2012 [28]	NR	NR
Borrell et al., 2017 [23]	Bilateral renal dysplasia	Arr 3p26.3(233,708–1,056,880x3,2,299,276)x2
	Hypospadias	Arr 2p25.1p24.2(10,064,304x2,11,291,005–17,228,290x1,18,714,949)x2

Abbreviations: ALSA: aberrant left subclavian artery, AVSD: atrioventricular septal defect, CCA: corpus callosum agenesis, CHD: congenital heart disease, CM: Cisterna Magna, CPC: chorioid plexus cysts, CSP: cavum septum pellucidum, NF: nuchal fold, NR: not reported, RAA: right aortic arch, SUA: single umbilical artery, VSD: ventricular septal defect.

## 4 Discussion

### 4.1 Principal Findings of Our Study

The study demonstrated, first, an overall of 3% incremental yield of CMA over karyotyping (95% CI 2%–5%, I^2^ = 0%) in FGR cases without any additional findings and, second, verified and reinforced the previous results of Borell et al. [9] by revealing an overall 4% incremental yield of CMA over karyotyping (95% CI 3%–5%, I^2^ = 0%) in nonmalformed FGR cases and an overall 10% incremental yield (95% CI –13%, I^2^ = 0%) in malformed FGR cases.

### 4.2 Comparison to Existing Literature

The findings of the present meta‐analysis are consistent with those of the earlier meta‐analysis by Borrell et al., which, based on data from 10 studies and a smaller sample size, demonstrated that the use of CMA in karyotypically normal fetuses with fetal growth restriction (FGR) yielded an incremental diagnostic yield of 4% (95% CI: 1%–6%) over conventional karyotyping in non‐malformed FGR fetuses, and 10% (95% CI: 6%–14%) in FGR fetuses with structural anomalies [9].

Chen X et al. reported that cardiovascular anomalies were the most frequently observed findings in fetuses with isolated structural abnormalities (33.3%), whereas increased nuchal translucency was the most common ultrasound soft marker (44.5%). Among fetuses with FGR, pathogenic or likely pathogenic copy number variants (P/LP CNVs) were detected in 6.7%, whereas no chromosomal aneuploidies or P/LP CNVs were found in cases with abnormal amniotic fluid volume [11].

Another study reported a higher diagnostic yield of CMA in FGR cases with structural anomalies (25%) compared to those with isolated FGR (9.6%) or non‐structural findings (6.8%), which are higher than the rates observed in our meta‐analysis. These elevated rates may be attributed to the inclusion of more severe or complex anomalies in that study population, which are more likely to be associated with chromosomal abnormalities [14]. In contrast to that study, we were not able to assess potential differences in the diagnostic yield between early‐ and late‐onset FGR due to the limited number of studies providing data stratified by timing of onset [14].

Our meta‐analysis is in agreement with individual studies supporting the view that chromosomal microarray analysis (CMA) is a reliable technique that enhances diagnostic yield in FGR fetuses compared to conventional karyotyping [6, 7, 12, 13, 17, 23, 26, 27, 29, 31]. According to Hui et al., this diagnostic yield may vary depending on the anatomical system involved [18]. Similarly, Dap et al. reported that the use of CMA improved the detection of genetic anomalies in fetuses with severe, isolated, and very early‐onset IUGR, with a diagnostic yield of 4.5%. These findings are consistent with the results of our meta‐analysis [15].

Both maternal and fetal age may influence the diagnostic yield of prenatal genetic testing. It is well established that the risk of fetal chromosomal abnormalities increases with advancing maternal age. Accordingly, the American College of Medical Genetics and Genomics (ACMG) recommends offering prenatal testing to women of advanced maternal age, as also supported by findings from Nguyen et al. [12, 32] However, our meta‐analysis was unable to explore this aspect because of the limited number of studies providing stratified data by maternal age.

Furthermore, De Wit et al. recommend offering chromosomal testing in fetuses diagnosed as small for gestational age between 18 and 24 weeks of gestation, which is consistent with the findings of Zhou et al., who suggested a similar approach in FGR fetuses under 32 weeks of gestation [16, 25]. As shown by Nguyen et al., this gestational window accounts for the majority of detected abnormalities, which may be attributed to the concurrent identification of structural anomalies during the second‐trimester detailed ultrasound [12]. They also emphasized the importance of postnatal clinical assessment and follow‐up, given that syndromic phenotypes with normal karyotypes are frequently encountered among SGA neonates [25].

However, Elron et al. investigated the diagnostic utility of genetic testing following amniocentesis in cases of late‐onset IUGR (> 28 weeks' gestation), supporting the value of CMA in late‐onset IUGR as well. They emphasized that third‐trimester ultrasound scans can reveal anomalies not detectable earlier in pregnancy, and in such cases, chromosomal microarray analysis may be valuable in elucidating the underlying genetic etiology [13].

Nevertheless, even after the extensive use of the conventional karyotype and CMA, a diagnosis remains elusive in numerous instances. As such, supplementary diagnostic modalities are essential to ascertain the genetic etiology of FGR and to offer the appropriate counseling and guidance. It is necessary to underline that the review and meta‐analysis conducted by Pauta et al. demonstrated an additional 12% incremental yield of Whole Exome Sequencing (WES) analysis in cases of isolated FGR with normal karyotype and CMA [33]. This additive value was further confirmed by two recent large studies in a similar [34] or even better [35] manner, potentially making WES analysis a valuable diagnostic asset not only for the investigation of congenital anomalies [36] but also for FGR and apparently idiopathic amniotic fluid abnormalities [37], findings that should, however, be further investigated and clinically assessed by larger studies.

### 4.3 Strengths and Limitations

To our knowledge, this is the first metanalysis that investigated the incremental yield of CMA over karyotyping in fetuses with isolated FGR and it is an update on the previous work conducted by Borell et al. on nonmalformed and malformed FGR cases. Our study analyzed data from 22 studies and included a total of 2275 fetuses that underwent CMA investigation after a normal karyotype analysis. Potential association of low ff and adverse pregnancy outcomes.

However, there are certain limitations to be noted. There is heterogeneity in the definition of fetal growth restriction since 4 different diagnostic modules were adopted across the included studies and 7 included studies did not report the FGR definition used. As such, it is evident that our results might differ if the recently published and adopted Delphi criteria were taken into consideration [38]. Furthermore, the inclusion criteria differed among the included studies, with some studies including all the growth restricted fetuses, others including FGR cases with additional structural anomalies or FGR cases with additional non‐structural findings, while others focused only on early‐onset cases of FGR. It is clear that the above mentioned variability may pose a selection bias risk in our study. Another limitation to be highlighted is the clinical design heterogeneity as in some studies CMA was undertaken subsequent to a normal karyotype or following normal FISH or QF‐PCR results while in others it was conducted concurrently with karyotyping. Additionally, the different sample sizes of the included cohorts as well as the utilization of various CMA platforms are parameters that should be taken into account in the interpretation of our results. Regarding the increased detection rates of submicroscopic CNVs in cases of malformed FGR, it should be underlined that the presence of several fetal abnormalities has been associated with underlying genetic aberrations [39, 40, 41]. As such, in the malformed cases, the additive effect of FGR in submicroscopic aberrations is yet unclear, given the already increased background risk due to the presence of complex structural abnormalities. Lastly, it should be noted that a subanalysis between early and late‐onset FGR was unable to be conducted as the studies demonstrated severe heterogeneity and the extraction of the necessary data was deemed unreliable. Some studies were considering early onset FGR only when the diagnosis took place before 24 weeks of gestational age [15, 19, 25] while Monier et al. did not report the total number of early or late onset FGR for us to be able to extract the incremental yield [17]. Furthermore, the study by Peng. et al. [20], while it divides the sample into early and late onset SGA, it does not provide the necessary data to make clear which of the fetuses with pathogenic CNVs were isolated, nonmalformed or malformed FGR. Eventually, there were only 2 studies that could be used for the reliable extraction and use of data that forced us to omit this subanalysis.

## 5 Conclusion

Our study demonstrated an overall 3% incremental yield of CMA over karyotyping in FGR cases without any additional findings, an overall 4% incremental yield of CMA in nonmalformed FGR cases and an overall 10% incremental yield in malformed FGR cases. These findings may be useful in clinical practice to guide management options and the counseling of the couples in an effort to individualize patient care and facilitate clinicians when they come across such a common clinical entity.

## Funding

The authors have nothing to report.

## Ethics Statement

The authors have nothing to report.

## Consent

The authors have nothing to report.

## Conflicts of Interest

The authors declare no conflicts of interest.

## Data Availability

Data sharing not applicable to this article as no datasets were generated or analyzed during the current study.

## References

[1] American College of Obstetricians and Gynecologists' Committee on Practice Bulletins—Obstetrics and the Society forMaternal‐FetalMedicin , “ACOG Practice Bulletin No. 204: Fetal Growth Restriction,” Obstetrics & Gynecology 133, no. 2 (February 2019): e97–e109: PMID: 30681542, 10.1097/AOG.0000000000003070. 30681542

[2] J. H. Francis , M. Permezel , and M. A. Davey , “Perinatal Mortality by Birthweight Centile,” Australian and New Zealand Journal of Obstetrics and Gynaecology 54, no. 4 (August 2014): 354–359: PMID: 24731210, 10.1111/ajo.12205. 24731210

[3] R. M. Silver , Y. Zhao , C. Y. Spong , et al., “Eunice kennedy Shriver National Institute of Child Health and Human Development Maternal‐Fetal Medicine Units (NICHD MFMU) Network. Prothrombin Gene G20210A Mutation and Obstetric Complications,” Obstetrics & Gynecology 115, no. 1 (January 2010): 14–20: PMID: 20027028; PMCID: PMC2981703, 10.1097/AOG.0b013e3181c88918. 20027028 PMC2981703

[4] Committee on Practice Bulletins—ObstetricsAmerican College of Obstetricians and Gynecologists , “Practice Bulletin No. 132: Antiphospholipid Syndrome,” Obstetrics & Gynecology 120, no. 6 (December 2012): 1514–1521: PMID: 23168789, 10.1097/01.AOG.0000423816.39542.0f. 23168789

[5] R. J. Snijders , C. Sherrod , C. M. Gosden , and K. H. Nicolaides , “Fetal Growth Retardation: Associated Malformations and Chromosomal Abnormalities,” American Journal of Obstetrics and Gynecology 168, no. 2 (February 1993): 547–555: PMID: 8438926, 10.1016/0002-9378(93)90491-z. 8438926

[6] F. Yue , M. Hao , D. Jiang , R. Liu , and H. Zhang , “Prenatal Phenotypes and Pregnancy Outcomes of Fetuses With 16p11.2 microdeletion/microduplication,” BMC Pregnancy and Childbirth 24, no. 1 (July 2024): 494: PMID: 39039444; PMCID: PMC11265082, 10.1186/s12884-024-06702-w. 39039444 PMC11265082

[7] Lovrecic L. , Remec Z. I. , Volk M. , Rudolf G. , Writzl K. , Peterlin B. “Clinical Utility of Array Comparative Genomic Hybridisation in Prenatal Setting.” BMC Medical Genetics 17, no. 1 (November 2016): 81. PMID: 27846804; PMCID: PMC5111187, 10.1186/s12881-016-0345-8. 27846804 PMC5111187

[8] R. J. Wapner , C. L. Martin , B. Levy , et al., “Chromosomal Microarray Versus Karyotyping for Prenatal Diagnosis,” New England Journal of Medicine 367, no. 23 (December 2012): 2175–2184: PMID: 23215555; PMCID: PMC3549418, 10.1056/NEJMoa1203382. 23215555 PMC3549418

[9] A. Borrell , M. Grande , M. Pauta , L. Rodriguez‐Revenga , and F. Figueras , “Chromosomal Microarray Analysis in Fetuses With Growth Restriction and Normal Karyotype: A Systematic Review and Meta‐Analysis,” Fetal Diagnosis and Therapy 44, no. 1 (2018): 1–9: Epub 2017 Sep 9. PMID: 28889126, 10.1159/000479506. 28889126

[10] K. O. Kagan , M. Hoopmann , and J. Sonek , “Second Trimester Soft Markers: Still Worth to be Mentioned?,” Archives of Gynecology and Obstetrics 311, no. 5 (May 2025): 1233–1240: Epub 2025 Apr 9. PMID: 40204923; PMCID: PMC12033118, 10.1007/s00404-025-08021-7. 40204923 PMC12033118

[11] X. Chen , L. Lan , H. Wu , et al., “Chromosomal Microarray Analysis in Fetuses With Ultrasound Abnormalities,” International Journal of General Medicine 17 (August 2024): 3531–3540: PMID: 39161407; PMCID: PMC11332413, 10.2147/IJGM.S472906. 39161407 PMC11332413

[12] P. T. Nguyen , T. N. L. Hoang , D. C. Tran , and Q. T. Nguyen , “The Yield of Using Single‐Nucleotide Polymorphism‐Based Chromosomal Microarray Analysis in Diagnosis the Genetic Etiology of Fetal Growth Restriction,” Clinica Terapeutica 176, no. 1 (January‐February 2025): 60–66: PMID: 39957452, 10.7417/CT.2025.5166. 39957452

[13] E. Elron , I. Maya , N. Shefer‐Averbuch , et al., “The Diagnostic Yield of Chromosomal Microarray Analysis in Third‐Trimester Fetal Abnormalities,” American Journal of Perinatology 41, no. 16 (December 2024): 2232–2242: Epub 2024 Apr 30. PMID: 38688298, 10.1055/s-0044-1786514. 38688298

[14] C. Peng , L. Hu , X. Bu , et al., “The Genetics and Clinical Outcomes in 151 Cases of Fetal Growth Restriction: A Chinese Single‐Center Study,” European Journal of Obstetrics & Gynecology and Reproductive Biology 298 (July 2024): 128–134: Epub 2024 May 11. PMID: 38756052, 10.1016/j.ejogrb.2024.05.004. 38756052

[15] M. Dap , F. Gicquel , L. Lambert , E. Perdriolle‐Galet , C. Bonnet , and O. Morel , “Utility of Chromosomal Microarray Analysis for the Exploration of Isolated and Severe Fetal Growth Restriction Diagnosed Before 24 Weeks' Gestation,” Prenatal Diagnosis 42, no. 10 (September 2022): 1281–1287: Epub 2022 Apr 18. PMID: 35426144, 10.1002/pd.6149. 35426144

[16] H. Zhou , K. Cheng , Y. Li , et al., “The Genetic and Clinical Outcomes in Fetuses With Isolated Fetal Growth Restriction: A Chinese Single‐Center Retrospective Study,” Frontiers in Genetics 13 (April 2022): 856522: PMID: 35571012; PMCID: PMC9096609, 10.3389/fgene.2022.856522. 35571012 PMC9096609

[17] I. Monier , A. Receveur , V. Houfflin‐Debarge , et al., “French Federation of Fetal Medicine Centers. Should Prenatal Chromosomal Microarray Analysis be Offered for Isolated Fetal Growth Restriction? A French Multicenter Study,” American Journal of Obstetrics and Gynecology 225, no. 6 (December 2021): 676.e1–676.e15: Epub 2021 May 29. PMID: 34058167, 10.1016/j.ajog.2021.05.035. 34058167

[18] A. S. Hui , M. H. K. Chau , Y. M. Chan , et al., “The Role of Chromosomal Microarray Analysis Among Fetuses With Normal Karyotype and Single System Anomaly or Nonspecific Sonographic Findings,” Acta Obstetricia et Gynecologica Scandinavica 100, no. 2 (February 2021): 235–243. Epub 2020 Oct 15. PMID: 32981064, 10.1111/aogs.14003. 32981064

[19] G. An , Y. Lin , L. P. Xu , et al., “Application of Chromosomal Microarray to Investigate Genetic Causes of Isolated Fetal Growth Restriction,” Molecular Cytogenetics 11, no. 1 (June 2018): 33. PMID: 29991965; PMCID: PMC5987400, 10.1186/s13039-018-0382-4. 29991965 PMC5987400

[20] R. Peng , J. Yang , H. N. Xie , M. F. Lin , and J. Zheng , “Chromosomal and Subchromosomal Anomalies Associated to Small for Gestational Age Fetuses With No Additional Structural Anomalies,” Prenatal Diagnosis 37, no. 12 (December 2017): 1219–1224. Epub 2017 Dec 3. Erratum in: Prenat Diagn. 2018 Feb;38(3):224. doi: 10.1002/pd.5218. Zhou, Yi [corrected to Yang, Jian‐Bo]. PMID: 29025195, 29025195 10.1002/pd.5169

[21] S. Brun , P. Pennamen , A. Mattuizzi , et al., “Interest of Chromosomal Microarray Analysis in the Prenatal Diagnosis of Fetal Intrauterine Growth Restriction,” Prenatal Diagnosis 38, no. 13 (December 2018): 1111–1119. Epub 2018 Nov 22. PMID: 30328630, 10.1002/pd.5372. 30328630

[22] M. Xia , X. Yang , J. Fu , Z. Teng , Y. Lv , and L. Yu , “Application of Chromosome Microarray Analysis in Prenatal Diagnosis,” BMC Pregnancy and Childbirth 20, no. 1 (November 2020): 696. PMID: 33198662; PMCID: PMC7667803, 10.1186/s12884-020-03368-y. 33198662 PMC7667803

[23] A. Borrell , M. Grande , E. Meler , et al., “Genomic Microarray in Fetuses With Early Growth Restriction: A Multicenter Study,” Fetal Diagnosis and Therapy 42, no. 3 (2017): 174–180. Epub 2016 Nov 2. Erratum in: Fetal Diagn Ther. 2017;42(3):240. doi: 10.1159/000458723. PMID: 27802431, 27802431 10.1159/000452217

[24] M. I. Srebniak , K. E. Diderich , M. Joosten , et al., “Prenatal SNP Array Testing in 1000 Fetuses With Ultrasound Anomalies: Causative, Unexpected and Susceptibility CNVs,” European Journal of Human Genetics 24, no. 5 (May 2016): 645–651: Epub 2015 Sep 2. PMID: 26328504; PMCID: PMC4930096, 10.1038/ejhg.2015.193. 26328504 PMC4930096

[25] M. C. de Wit , M. I. Srebniak , M. Joosten , et al., “Prenatal and Postnatal Findings in Small‐for‐Gestational‐Age Fetuses Without Structural Ultrasound Anomalies at 18‐24 Weeks,” Ultrasound in Obstetrics and Gynecology 49, no. 3 (March 2017): 342–348: PMID: 27102944, 10.1002/uog.15949. 27102944

[26] H. Zhu , S. Lin , L. Huang , et al., “Application of Chromosomal Microarray Analysis in Prenatal Diagnosis of Fetal Growth Restriction,” Prenatal Diagnosis 36, no. 7 (July 2016): 686–692: Epub 2016 Jun 21. PMID: 27221052, 10.1002/pd.4844. 27221052

[27] B. Oneda , R. Baldinger , R. Reissmann , et al., “High‐Resolution Chromosomal Microarrays in Prenatal Diagnosis Significantly Increase Diagnostic Power,” Prenatal Diagnosis 34, no. 6 (June 2014): 525–533: Epub 2014 Mar 21. PMID: 24919595, 10.1002/pd.4342. 24919595

[28] L. G. Shaffer , J. A. Rosenfeld , M. P. Dabell , et al., “Detection Rates of Clinically Significant Genomic Alterations by Microarray Analysis for Specific Anomalies Detected by Ultrasound,” Prenatal Diagnosis 32, no. 10 (October 2012): 986–995: Epub 2012 Jul 30. PMID: 22847778; PMCID: PMC3509216, 10.1002/pd.3943. 22847778 PMC3509216

[29] N. Gruchy , M. Decamp , N. Richard , et al., “Array CGH Analysis in High‐Risk Pregnancies: Comparing DNA From Cultured Cells and Cell‐Free Fetal DNA,” Prenatal Diagnosis 32, no. 4 (April 2012): 383–388: Epub 2011 Oct 24. PMID: 22025315, 10.1002/pd.2861. 22025315

[30] L. Kleeman , D. W. Bianchi , L. G. Shaffer , et al., “Use of Array Comparative Genomic Hybridization for Prenatal Diagnosis of Fetuses With Sonographic Anomalies and Normal Metaphase Karyotype,” Prenatal Diagnosis 29, no. 13 (December 2009): 1213–1217: PMID: 19862770; PMCID: PMC4459708, 10.1002/pd.2367. 19862770 PMC4459708

[31] I. B. Van den Veyver , A. Patel , C. A. Shaw , et al., “Clinical Use of Array Comparative Genomic Hybridization (aCGH) for Prenatal Diagnosis in 300 Cases,” Prenatal Diagnosis 29, no. 1 (January 2009): 29–39: PMID: 19012303; PMCID: PMC3665952, 10.1002/pd.2127. 19012303 PMC3665952

[32] F. S. Collins , “Shattuck Lecture‐‐Medical and Societal Consequences of the Human Genome Project,” New England Journal of Medicine 341, no. 1 (July 1999): 28–37: PMID: 10387940, 10.1056/NEJM199907013410106. 10387940

[33] M. Pauta , R. J. Martinez‐Portilla , E. Meler , J. Otaño , and A. Borrell , “Diagnostic Yield of Exome Sequencing in Isolated Fetal Growth Restriction: Systematic Review and Meta‐Analysis,” Prenatal Diagnosis 43, no. 5 (May 2023): 596–604: Epub 2023 Mar 25. PMID: 36869857, 10.1002/pd.6339. 36869857

[34] X. Shi , Y. Huang , H. Ding , L. Zhao , W. He , and J. Wu , “Utility of Whole Exome Sequencing in the Evaluation of Isolated Fetal Growth Restriction in Normal Chromosomal Microarray Analysis,” Annals of Medicine 57, no. 1 (December 2025): 2476038: Epub 2025 Mar 11. PMID: 40066675; PMCID: PMC11899204, 10.1080/07853890.2025.2476038. 40066675 PMC11899204

[35] Y. Chen , M. Cai , M. Chen , et al., “Detection of Chromosomal and Gene Abnormality With Karyotyping, Chromosomal Microarray Analysis and Trio‐Based Whole Exome Sequencing in Pregnancies With Fetal Growth Restriction: Implications for Precise Prenatal Diagnosis,” BMC Pregnancy and Childbirth 25, no. 1 (October 2025): 1067: PMID: 41073961; PMCID: PMC12513148, 10.1186/s12884-025-08164-0. 41073961 PMC12513148

[36] J. Lord , D. J. McMullan , R. Y. Eberhardt , et al., “Prenatal Exome Sequencing Analysis in Fetal Structural Anomalies Detected by Ultrasonography (PAGE): A Cohort Study,” Lancet 393, no. 10173 (February 2019): 747–757: Epub 2019 Jan 31. PMID: 30712880; PMCID: PMC6386638, 10.1016/S0140-6736(18)31940-8. 30712880 PMC6386638

[37] N. Grausz , M. V. Senat , C. Colmant , A. Boizard , A. Benachi , and H. Bouchghoul , “Idiopathic Polyhydramnios and Postnatal Outcomes of Children: The Role of Exome Sequencing,” Prenatal Diagnosis 44, no. 11 (October 2024): 1279–1287: Epub 2024 Apr 29. PMID: 38682787, 10.1002/pd.6573. 38682787

[38] S. J. Gordijn , I. M. Beune , B. Thilaganathan , et al., “Consensus Definition of Fetal Growth Restriction: A Delphi Procedure,” Ultrasound in Obstetrics and Gynecology 48, no. 3 (September 2016): 333–339: PMID: 26909664, 10.1002/uog.15884. 26909664

[39] R. Corroenne , D. Paladini , I. Papastefanou , et al., “Prenatal Evaluation, Diagnosis and Management of Fetal Corpus Callosal Abnormalities: International Delphi Consensus,” Ultrasound in Obstetrics and Gynecology 66, no. 5 (August 2025): 582–588: Epub ahead of print. PMID: 40847729, 10.1002/uog.70003. 40847729 PMC12579776

[40] Q. Lu , L. Luo , B. Zeng , et al., “Prenatal Chromosomal Microarray Analysis in a Large Chinese Cohort of Fetuses With Congenital Heart Defects: A Single Center Study,” Orphanet Journal of Rare Diseases 19, no. 1 (August 2024): 307: PMID: 39175064; PMCID: PMC11342572, 10.1186/s13023-024-03317-4. 39175064 PMC11342572

[41] R. Huang , F. Fu , H. Zhou , et al., “Prenatal Diagnosis in the Fetal Hyperechogenic Kidneys: Assessment Using Chromosomal Microarray Analysis and Exome Sequencing,” Human Genetics 142, no. 6 (June 2023): 835–847: Epub 2023 Apr 24. PMID: 37095353, 10.1007/s00439-023-02545-1. 37095353

